# Biotechnology revival: *in situ* sludge minimization in wastewater

**DOI:** 10.3389/fmicb.2025.1603215

**Published:** 2025-05-02

**Authors:** Yiqiang Chen, Xu Jiang, Maosheng Yang, Zhu Wang

**Affiliations:** ^1^Institute of Environmental Research at Greater Bay, Key Laboratory for Water Quality and Conservation of the Pearl River Delta, Ministry of Education, Guangzhou University, Guangzhou, China; ^2^Key Laboratory of Environmental Remediation and Ecological Health, Ministry of Industry and Information Technology, Jiangsu Environmental Engineering Technology Co., Ltd, Jiangsu Environmental Protection Group Co., Ltd, Nanjing, China; ^3^Key Laboratory of Integrated Regulation and Resources Development on Shallow Lakes, Ministry of Education, College of Environment, Hohai University, Nanjing, China

**Keywords:** *in situ* sludge reduction, environmental biotechnology, bacteriophage lysis, carbon emission, carbon neutrality

## Abstract

In the face of the escalating challenge of sludge production and disposal in wastewater treatment plants (WWTPs), *in situ* sludge reduction biotechnology (ISRB) has recently emerged as a highly promising strategy. It not only has the potential to curtail sludge generation at its origin but also ensures the sustained efficiency of the treatment process. Several key strategies have demonstrated exceptional potential in harnessing microbial processes for sludge degradation. They encompass enzymatic hydrolysis, microbial inoculation, protozoan/metazoan predation, bacteriophage lysis, and biofilm-based manipulation. Compared to traditional methods (e.g., incineration and landfilling), these biotechnologies offer significant advantages through lower costs, reduced energy consumption, and minimal environmental impacts. The efficacy of ISRB is substantially affected by various factors, where pH, microbial shift, and nutrient conditions play crucial roles. Despite the notable progress made in this field, significant challenges persist when it comes to scaling up these technologies for more extensive and widespread applications. This review comprehensively highlights the fundamental mechanisms, application strategies, and future prospects of ISRB, including one of the first studies to introduce bacteriophage-based approaches for *in situ* sludge reduction, offering a novel perspective on phage-mediated sludge control. By doing so, it aims to offer in-depth insights into the role of ISRB as a sustainable solution for sludge management, paving the way for further research and development in this crucial area of environmental biotechnology.

## 1 Introduction

**Figure 1:**
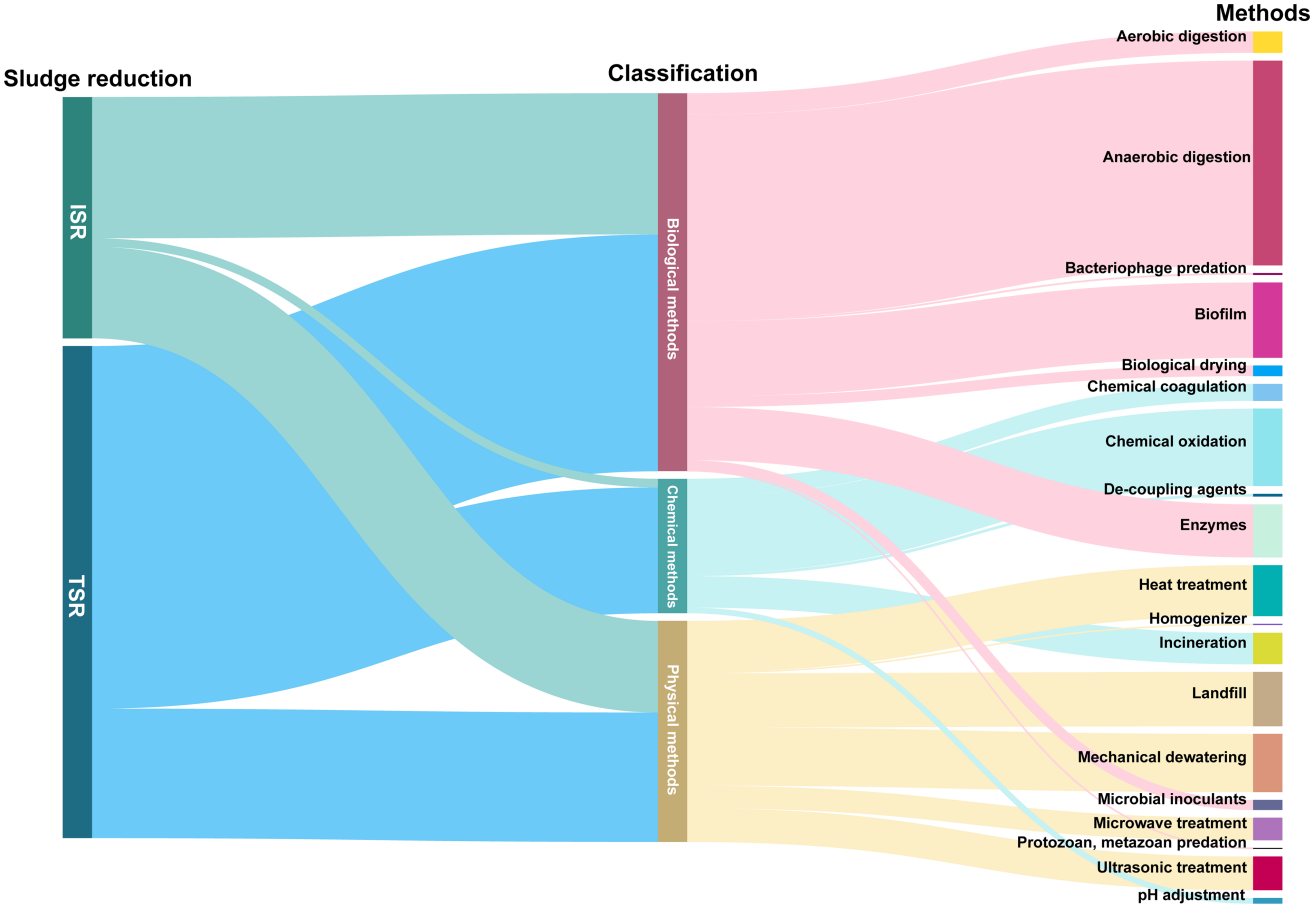
Theme distribution of published studies on sludge reduction methods in the Web of Science database.

Currently, it is estimated that global WWTPs generate more than 103 million tons of waste activated sludge (WAS) on a wet-matter basis (Li L. et al., 2023). WAS can be a reservoir of various contaminants, including heavy metals, organic pollutants, and pathogens (Hua et al., 2025; Du and Qiu, 2024). Improper management of WAS can lead to serious pollution of soil, water, and atmosphere, which in turn could pose significant risks to both human health and ecological safety (Newell et al., 2024; Khan et al., 2023). In response to these challenges, countries worldwide have been actively implementing strategies to curtail sludge production. For instance, China has set an ambitious target to promote the reduction and recycling of WAS by the end of 2025, aiming to achieve a disposal rate of over 90% (Zhu et al., 2025). At the EU level, the Council Directive 86/278/EEC promoted the safe utilization of sludge and enhances resource efficiency through nutrient recovery (Valchev et al., 2024). Sludge reduction strategies can be broadly classified into two categories, as shown in Figure 1, traditional sludge reduction (TSR), often regarded as end-of-pipe treatment, and *in situ* sludge reduction (ISR) (Zhou et al., 2023). In contemporary times, developed nations such as the United States have adopted TSR techniques and achieved comparatively high efficiency levels in sludge reduction. However, TSR methods such as incineration and landfilling are fraught with environmental drawbacks.

These processes are known to trigger secondary pollution, manifested in multiple forms. For instance, they contribute to greenhouse gas emissions, which are a major driver of global climate change (Chu et al., 2024). In addition, the formation of dioxins, highly toxic and persistent organic pollutants, poses a significant threat to both human health and the ecosystem (Li X. et al., 2023). Heavy metal leakage from landfilled sludge can contaminate soil and groundwater, further exacerbating environmental degradation (Ma G. et al., 2023). Another critical concern associated with TSR is its substantial financial burden. Studies have reported that 50–60% of the operational costs of WWTPs are allocated to sludge reduction and disposal (Appels et al., 2008; Campos et al., 2009; Wang et al., 2011). This high cost not only strains the economic resources of WWTPs but also limits the long-term sustainability of these treatment facilities.

**Figure 2:**
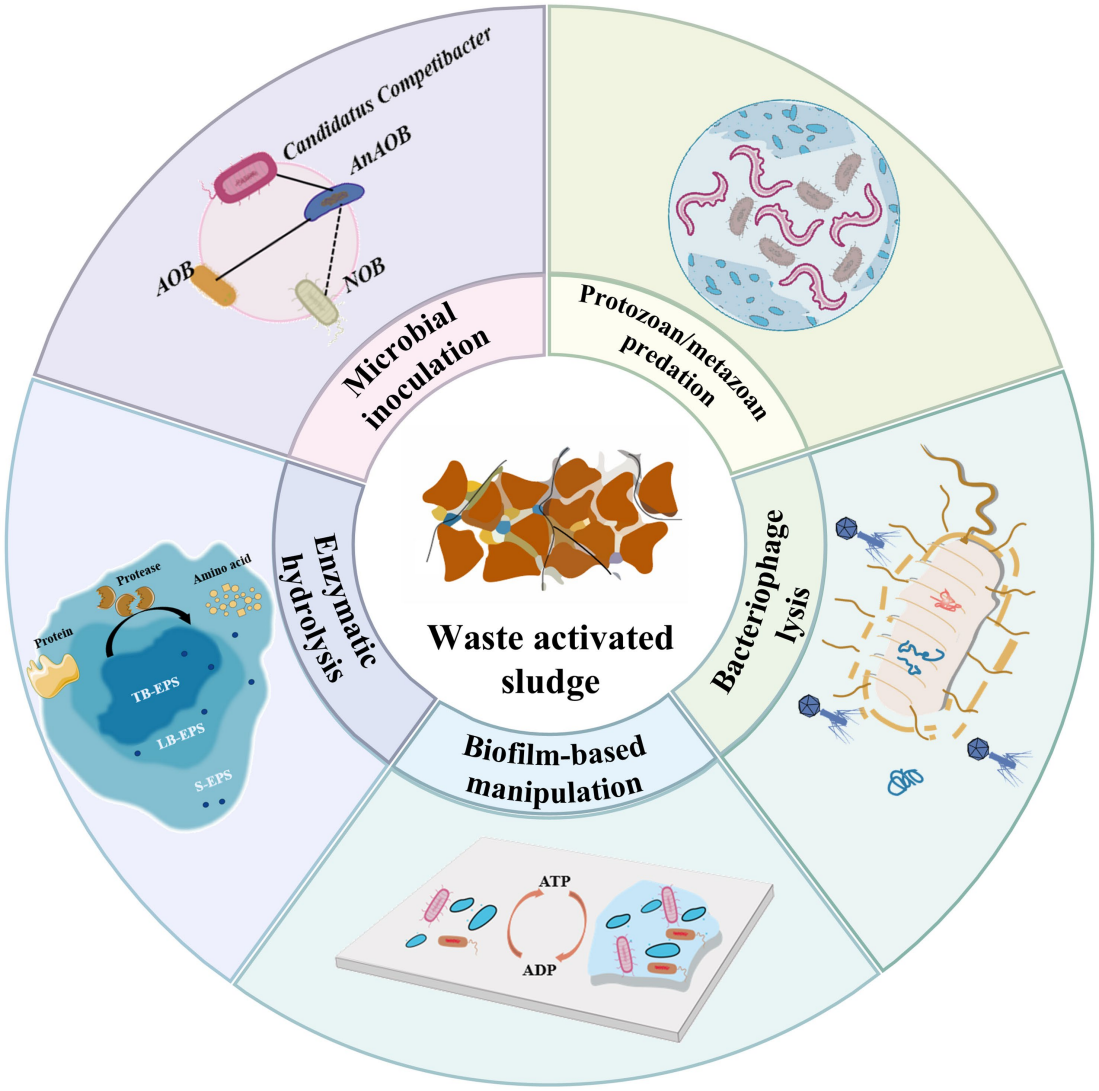
Overview of *in situ* sludge reduction biotechnologies.

Therefore, the environmental and economic drawbacks of TSR call for the exploration and implementation of more sustainable alternatives, such as ISR methods, to reduce the WAS on site (Mu et al., 2021). ISR endeavors to curtail the generation of WAS while maintaining effluent water quality, which redirects the degradation pathway of organic matter from promoting microbial proliferation toward thermal dissipation (Guo et al., 2013). One hundred years ago, Ardern and Lockett (Sheik et al., 2014) invented activated sludge method to biologically treatment wastewater. Interestingly, now researchers are revisiting biotechnology as a new strategy to minimize sludge reduction (Wang K. et al., 2024; Sudharsan et al., 2023). When juxtaposed with physicochemical methods for ISR, biotechnological methods could harness indigenous microbes to break down organic substances in excess sludge, offering exclusive advantages such as cost-effectiveness, low energy consumption, minimal environmental risk, and ease of subsequent recycling (Wang Y. et al., 2023; Li W.S. et al., 2023; Thakur et al., 2024). Nevertheless, research on ISRB remains in its nascent stage and demands further exploration. Previous studies have primarily focused on one of the following aspects: (i) Enzymatic hydrolysis: A combination of cellulase, protease, and lipase enzymes were found effective for sludge reduction, which could diminish the organic component of biosolids by approximately 70% (Nalladiyil et al., 2023). (ii) Microbial inoculation: Hydrolytic bacteria (*Candidatus_Competibacter*) could break down large organic molecules to reduce the organic content in sludge, while predatory bacteria (*Norank_f__Saprospiraceae*) could facilitate cell lysis through predation, releasing intracellular materials (Peng et al., 2023). In addition, slow-growing bacteria (*Azospira*) could reduce sludge production through metabolic uncoupling; and fermentative bacteria (*Anaerolineaceae*) could further decompose organic matter via fermentation to achieve sludge reduction (Bian et al., 2020; Fan et al., 2023). (iii) Protozoan/metazoan predation: Protozoa (Ciliophora, Oligohymenophorea, Vermamoeba vermiformis) (Miwa et al., 2024) and metazoa (Oligochaete, Nematode, Rotifer) (Gad et al., 2024) could reduce biomass growth by preying on excess bacteria, thereby improving sludge settling performance. (iv) Bacteriophage lysis: Phage GTE7 can lyse bacteria of the genera *Gordonia* and *Nocardia*, thereby stabilizing foam. Phage HHY could target the filamentous bacterium *Haliscomenobacter hydrossis* responsible for sludge bulking, leading to an increased sludge settling rate and a reduced sludge volume index (Ragab et al., 2024; Shivaram et al., 2023). (v) Biofilm-based manipulation: Sludge reduction can be achieved through the synergistic effects of sludge fermentation, microbial enrichment in biofilms, and the reduction of suspended sludge discharge (Fan et al., 2023; Zhao et al., 2023; Riechelmann et al., 2024), as shown in Figure 2.

As far as we know, there have been very limited studies summarizing the advancements of abovementioned five aspects, and even fewer have delved into the impact of bacteriophages on *in situ* sludge reduction. This study endeavors to showcase the latest progress of ISRB in wastewater treatment systems. It comprehensively presents the unique merits, underlying mechanisms, practical application strategies, and future prospects of ISRB, offering novel perspectives for the eco-friendly treatment of surplus sludge.

## 2 Exclusive advantages of ISRB

### 2.1 TSR and ISR

**Figure 3:**
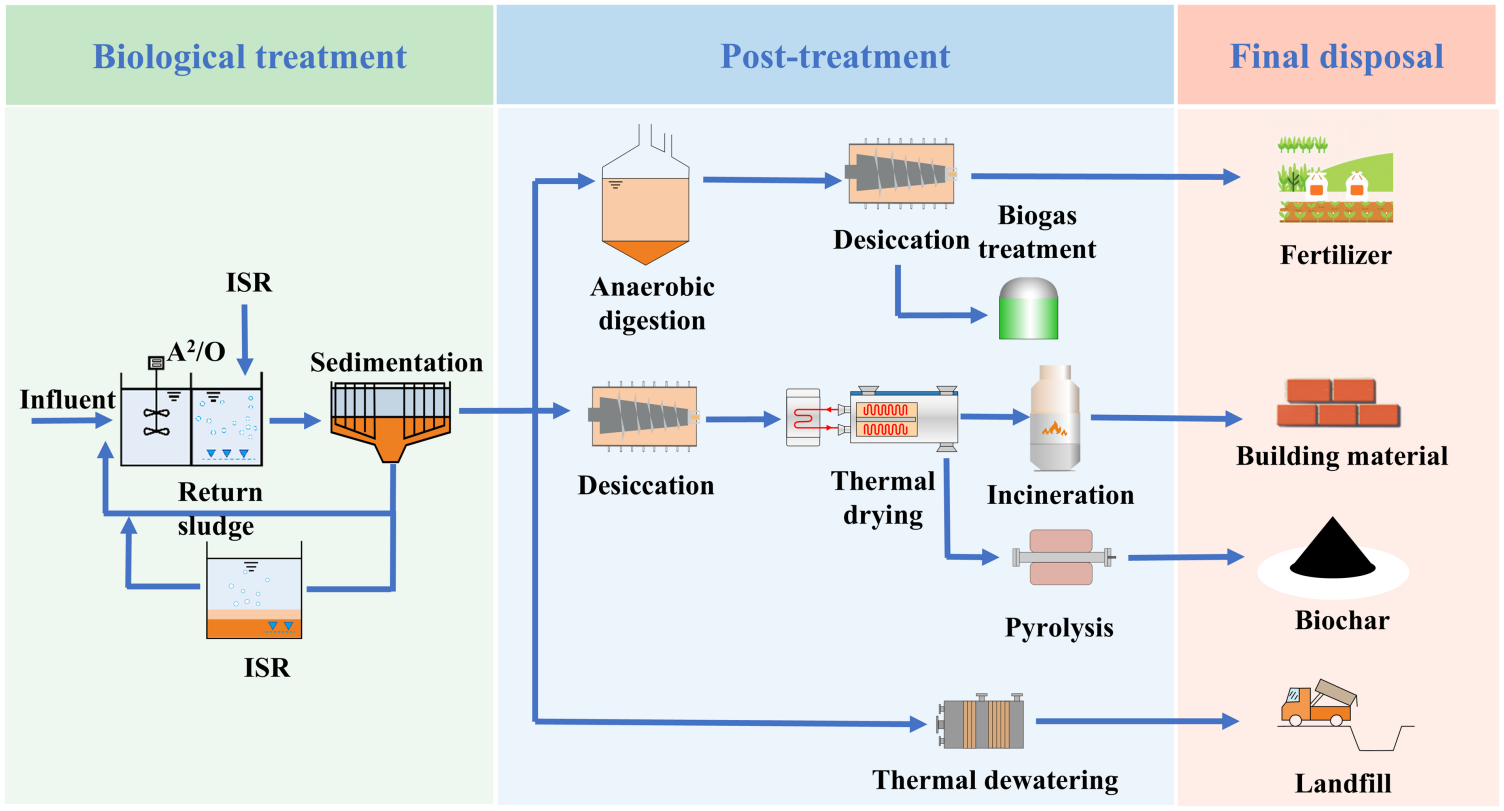
Commonly applied TSR and ISR procedures in wastewater systems.

Figure 3 illustrated the common process flow for sludge reduction treatment. TSR methods primarily encompass incineration, landfilling, and anaerobic digestion, all of which entail substantial fixed-asset expenditures. Incineration is a method that can efficiently diminish sludge volume through the complete oxidation of organic matter. However, to minimize air pollution, it necessitates investment in state-of-the-art emission control technologies (Liu et al., 2023). Landfilling has faced limitations in its application due to its long-term adverse environmental impacts on the soil. The leachate generated from landfilled sludge can contaminate groundwater, while the decomposition of organic matter in landfills leads to significant greenhouse gas emissions (Zhao et al., 2019). Anaerobic digestion, on the other hand, demands a considerable amount of thermal energy to maintain the ideal temperature for the anaerobic microorganisms to function effectively. Even after the digestion process, the residual sludge still has a high moisture content, which requires additional treatment steps (Wang et al., 2022). Overall, each of these TSR methods has its own drawbacks in terms of cost, environmental impact, and post-treatment requirements, highlighting the need for more sustainable and efficient sludge management strategies.

The advent of ISR methods presents a promising solution to the issues mentioned above, which has consequently captured global attention (Cheng et al., 2021; Wang et al., 2019; Zhou et al., 2023). ISR methods are designed to curtail sludge production at its source during the generation process, thereby effectively alleviating the burden and associated costs of subsequent sludge handling. In the treatment process, the volume of WAS has been minimized, and the removal efficiency was significantly enhanced by meticulously adjusting biological, chemical, or physical conditions (Li Y.L. et al., 2022; Li C. et al., 2022; Li W. et al., 2022). Specifically, traditional ISR methods encompass multiple strategies. One approach involves precisely controlling the microbial oxygen consumption. By regulating the oxygen supply, the metabolic activities of microorganisms can be optimized, which in turn affects the growth and decomposition of sludge. Another important measure is to reduce the usage of chemical coagulants. Excessive use of chemical coagulants can lead to increased sludge production and potential environmental risks. Minimizing their use not only cuts down on costs but also promotes a more sustainable treatment process. Furthermore, advanced oxidation techniques, such as ozone or ultrasonic treatment, have been employed. These techniques are capable of breaking down complex organic substances, enhancing the biodegradability of the sludge (Apollo et al., 2023). The improvement in biodegradability facilitates the subsequent treatment steps and ultimately contributes to a more efficient sludge reduction and removal process.

The implementation of ISR method demonstrated profound environmental and economic implications. By leveraging advanced biological and physical–chemical processes, ISR can effectively curtail sludge production, mitigate the potential for secondary pollution and offer notable economic advantages. The adoption of ISR can lead to a remarkable improvement in the operational efficiency of WWTPs and align seamlessly with the overarching goals of sustainable development (Wang L. et al., 2024).

### 2.2 ISRB and physicochemical counterparts

In comparison to ISRB, physicochemical methods exhibit pronounced drawbacks. Physical treatment techniques such as ultrasonic and microwave treatment primarily rely on mechanical shear, cavitation effects, or localized high temperatures and pressures to disrupt sludge flocs and lyse microbial cells (Parvathy Eswari et al., 2022). These processes can effectively reduce sludge volume or enhance subsequent anaerobic digestion efficiency. However, the substantial energy input required for these treatments can significantly increase the operational costs of WWTPs and considerably amplify their carbon footprint (Shen et al., 2022). Chemical methods, for instance, the addition of coagulants, flocculants, or oxidizing agents, achieve rapid sludge thickening and volume reduction by altering the surface charge, structural stability, and water-binding characteristics of sludge particles. While effective in the short term, these methods often result in the accumulation of chemical residues (Guo et al., 2024). These residues may enter aquatic ecosystems through treated water discharge, posing potential threats to the ecological balance, biodiversity, and overall health of these ecosystems (Kong et al., 2023). In addition, when evaluating the economic viability of physicochemical methods, one must take into account both the initial investment and subsequent maintenance costs. The high-cost equipment, continuous energy consumption, and the need for a steady supply of chemicals could offset the overall economic benefits of these methods, making them less attractive from a long-term financial perspective (Liu et al., 2022).

Conversely, ISRB could offer a more sustainable alternative. Strategies such as the addition of enzymes, microbial agents and bacteriophage cocktails can effectively reduce WAS production on-site. These approaches harness specific biological processes to achieve sludge reduction (Yin et al., 2020). By artificially enhancing the biodegradation capacity, ISRB could minimize the reliance on chemical inputs and energy-intensive operations, leading to a reduction in greenhouse gas emissions and aligning with the principles of green and sustainable wastewater treatment (Keb-Fonseca et al., 2021). Table 1 summarized ISR methods, highlighting the mechanisms, advantages, disadvantages, and sludge reduction rates of various methods.

**Table 1:** Comparison of different ISR methods.

Methods	Mechanism	Advantages	Disadvantages	Sludge reduction rate
Physical methods	Heat, microwave, Ozone, etc.	Efficient and fast	High energy consumption	35.1–43% (Sun et al., 2020; Ding and Jiang, 2013)
	Mechanical crushing, ultrasound, etc.	Low reaction time	Limited efficiency, maintenance issue	17.6–78% (Zhang et al., 2021; Wang Y. et al., 2021)
Chemical methods	NaOH, advanced oxidation, etc.	Kill pathogens	Expensive, secondary pollution	27–77% (Chiavola et al., 2021)
	De-coupling	Relatively simple, low cost	Toxicity and sludge bulking	80% (Fang et al., 2015)
ISRBs	Enzymatic hydrolysis	Low cost, low energy consumption, low environmental risk, relatively simple	Stability to be verified	30–50% (Parmar et al., 2001)
	Microbial inoculation			12–58.4% (Bian et al., 2020; Merrylin et al., 2013)
	Protozoan/metazoan predation			33–65% (Navaratna et al., 2014; Li et al., 2019)
	Bacteriophage lysis			33% (SVI) (Ragab et al., 2024)
	Biofilm-based manipulation			27.3–72.1% (Fan et al., 2023; Wang et al., 2018)

### 2.3 Sludge reduction and carbon emissions

WWTPs are significant contributors to carbon emissions, accounting for approximately 2% of the total societal carbon footprint. If all WASs were treated via incineration, this could lead to the generation of over 7,023 tons of carbon dioxide equivalent (tCO_2_-eq) (Zhang et al., 2022). ISRB could offer an effective approach to indirectly mitigate these carbon emissions by curbing the volume of sludge produced. When calculated based on an 80% sludge reduction rate achieved through ISRB, subsequent sludge disposal methods such as anaerobic digestion result in a substantially lower carbon emission of merely 576 tCO_2_-eq, as shown in Table 2.

**Table 2:** Cost and carbon emissions of different sludge reduction methods.

Methods	Cost (USD/t)	Carbon emissions (tCO_2_-eq)
Landfilling	276 (Kacprzak et al., 2017)	4,761 (Wei et al., 2020)
Anaerobic digestion	95.5 (Yang et al., 2015)	2,880 (Wei et al., 2020)
Incineration	67–70 (Panepinto et al., 2016)	7,023 (Wei et al., 2020)
ISRB	20 (Bian et al., 2020)	576

Compared with physicochemical methods, ISRB can significantly reduce carbon emissions through the following “3R” strategies (Guo et al., 2013):

- Energy consumption reduction: It is well-established that sludge transportation, incineration, and landfilling are energy-intensive processes. By minimizing the amount of sludge, ISRB reduces the energy demand associated with these processes (Zhao et al., 2019).
- Reconfiguration of external chemical agents: Avoiding the use of sludge dewatering agents can cut down on the carbon emissions associated with their upstream production processes. ISRB presents an alternative that circumvents the need for such agents (Yu et al., 2024).
- Reinforcement of removal efficiency: Through the regulation of the microbial community, ISRB can enhance the biodegradability of sludge and optimize metabolic pathways, thereby leading to a decrease in greenhouse gas emissions during the treatment process (Guo et al., 2013).

## 3 ISRB mechanisms

### 3.1 Enzymatic hydrolysis

**Figure 4:**
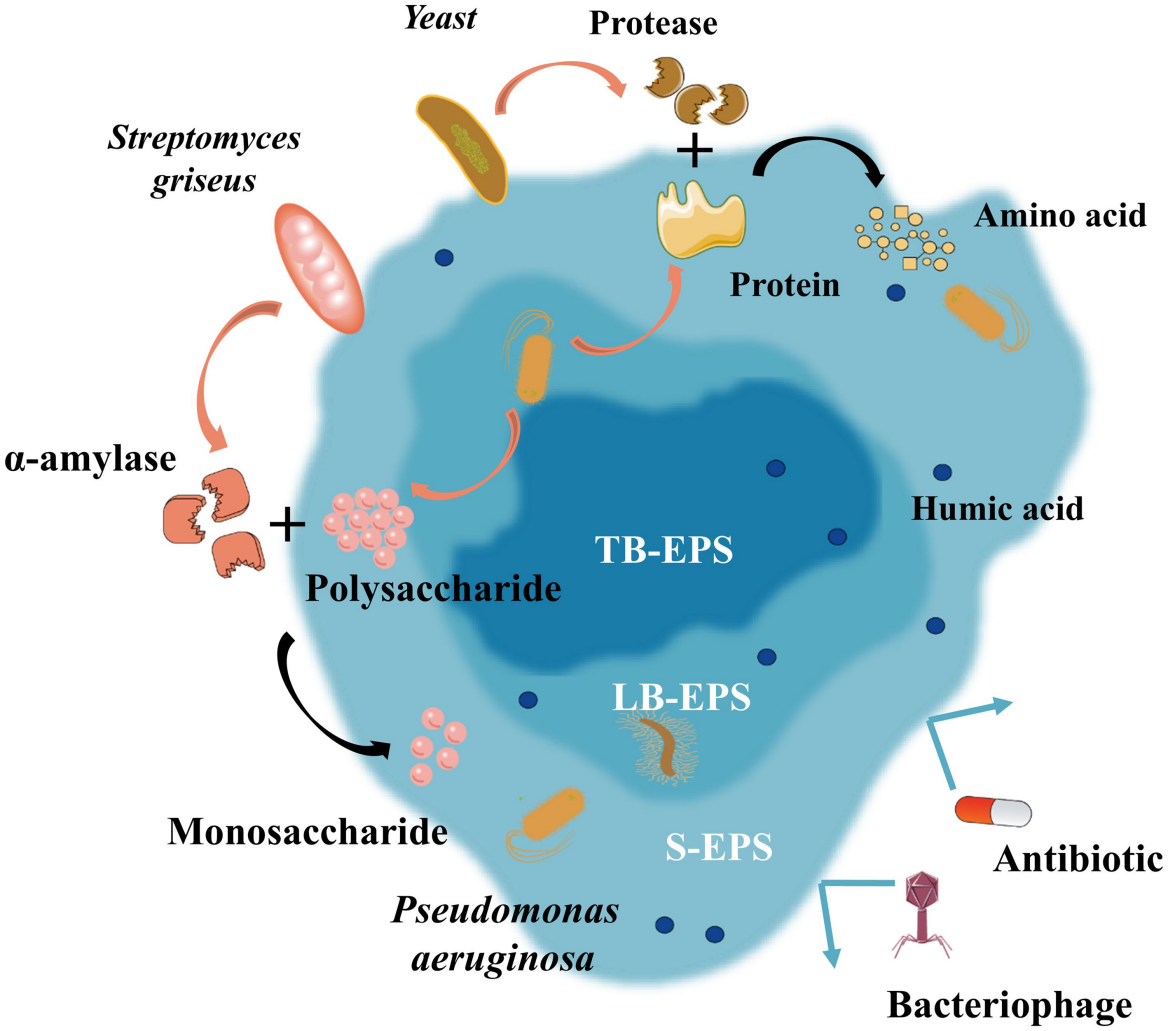
ISRB mechanisms of enzymatic hydrolysis.

Enzymes could break down complex organic molecules, thereby facilitating the reduction of WAS and enhancing its biodegradability (Sharma and Leung, 2021). The biotechnological mechanism underlying enzyme-mediated ISR primarily encompasses the following key aspects, as shown in Figure 4:

- Hydrolysis of extracellular polymeric substances (EPS): EPS serve as a protective shield for diverse microorganisms, acting as the initial line of defense during the process of excess sludge lysis (Flemming, 2016). Recent investigations have revealed that alpha-amylase derived from *Streptomyces griseus* could effectively disrupt the EPS of multidrug-resistant bacteria, with a particular efficacy against *Pseudomonas aeruginosa*. In the case of *P. aeruginosa*, the total carbohydrate content within the EPS matrix was notably reduced to 74.09% (Lakshmi et al., 2022). This hydrolysis of EPS has far-reaching implications for the rheological properties of the sludge and, consequently, the stability of biofilms (Kanwar et al., 2019).
- Cell lysis and cryptic growth: Enzyme-based pretreatment methods have been compellingly demonstrated to substantially enhance sludge solubilization and curtail suspended solids, with reductions of up to 22 and 17.14% achieved, respectively. Enzyme-induced cell lysis could trigger the liberation of intracellular substances, such as phosphorus and nitrogen. These released nutrients can be assimilated by symbiotic microorganisms through a process referred to as cryptic growth. This mechanism could facilitate nutrient cycling and play a pivotal role in biomass reduction, thereby significantly augmenting the overall efficiency of ISR (Preethi Banu et al., 2022).
- Alteration of sludge dewaterability: A synergistic mixture of protease, β-glucanase, cellulase, lipase, and α-amylase has been shown to remarkably improve the dewaterability of sludge (Nesterov et al., 2024; Wang C. et al., 2024). When introduced into the anaerobic digester, this enzyme blend resulted in a notable decline in the CST, from 827 s to 755 s. This treatment could enhance the sludge dewaterability and hold great promise for optimizing the overall efficiency of anaerobic digestion processes. Consequently, it presents a highly promising strategy for effective sludge management (Tas et al., 2018).

### 3.2 Microbial inoculation

Microbial inoculants could modulate and optimize the microbial community structure within sludge treatment systems, which serves multiple crucial functions, including the reduction of biomass production, enhancement of system stability, and improvement of removal efficiency, ultimately leading to the achievement of sludge reduction goals. However, to ensure the long-term effectiveness and sustainability of this process, precise microbial selection and well-thought-out system management strategies are indispensable.

- Introduction of slow-growing microorganisms as dominant species: The enrichment of ammonia-oxidizing bacteria and anaerobic ammonia oxidizing bacteria has been effectively implemented in the SNADF system, where they were characterized by a low growth rate and high efficiency in converting ammonia nitrogen, consequently leading to a reduction in the total amount of sludge (Ma L. et al., 2023). Remarkably, the denitrification efficiency of the system can reach up to 94.6%, with the observed yield coefficient as low as 0.05 kgMLSS/kgCOD (Zhang Y. et al., 2023). Additionally, optimizing operational parameters, such as employing aerobic granular sludge process, anaerobic feeding, periodic feast-famine cycling, and shortened settling durations, can enhance sludge granulation. Meanwhile, the selection of slow-growing microorganisms with unique metabolic traits further advances this granulation process (Nancharaiah and Sarvajith, 2019).
- Inhibition of high growth rate species: Introducing bacteria from the *Bacillus* genus, which are capable of producing antibiotics and exoenzymes, can effectively suppress the growth of other rapidly growing microorganisms, such as *V. vulnificus*. This suppression helps in inhibiting sludge accumulation. For instance, You et al. (Yaylacı, 2021) reported that when *B. pumilus* PJ_11 culture was inoculated at a level of 1.0 × 10^8^ CFU/mL, the growth of *V. vulnificus* was inhibited by 58.1% after 120 h.
- Optimization of microbial composition: The utilization of specific microbial species, such as *Acetobacter* and *Syntrophomonas*, into anaerobic membrane bioreactors has been demonstrated to enhance the hydrolysis and acetogenesis of primary sludge (Amha et al., 2019). Compared to non-bioaugmented operation, bioaugmentation has shown significant improvements, which could increase the overall hydrolysis by 38%, elevate the acetic acid levels in the acid-phase by 105%, and reduce solids by 55% (Martin-Ryals et al., 2020). On one hand bioaugmentation strengthens the system’s adaptability to environmental changes, on the other hand it enhances the synergistic interactions among different microbes, which in turn optimizes the degradation of organic matter and nutrient removal (Gao et al., 2024).

**Figure 5:**
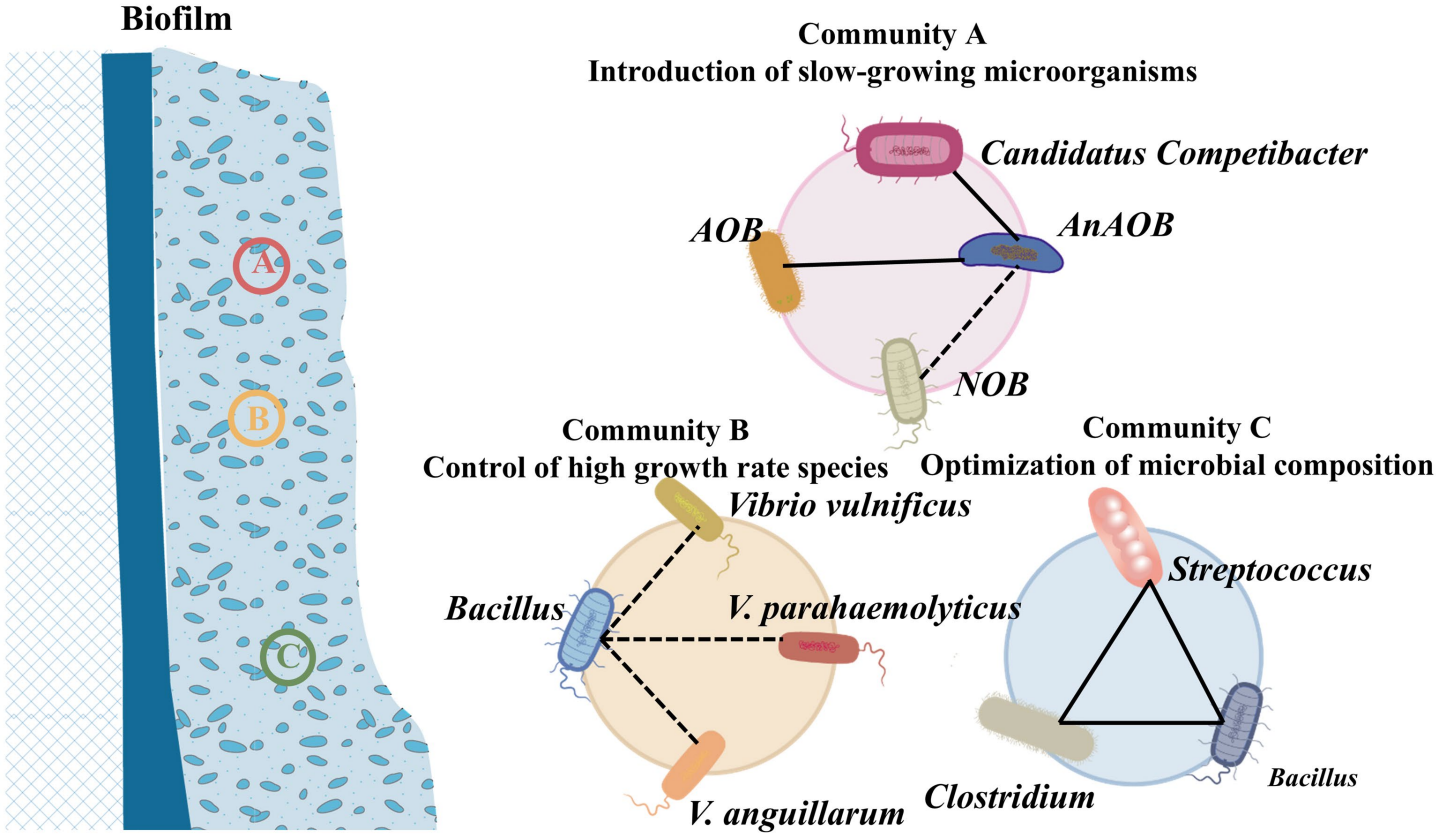
ISRB mechanisms of microbial inoculation.

Collectively, these strategies could significantly elevate the wastewater treatment system’s efficiency and minimize sludge production (Zhou et al., 2024), as shown in Figure 5.

### 3.3 Protozoan/metazoan predation

**Figure 6:**
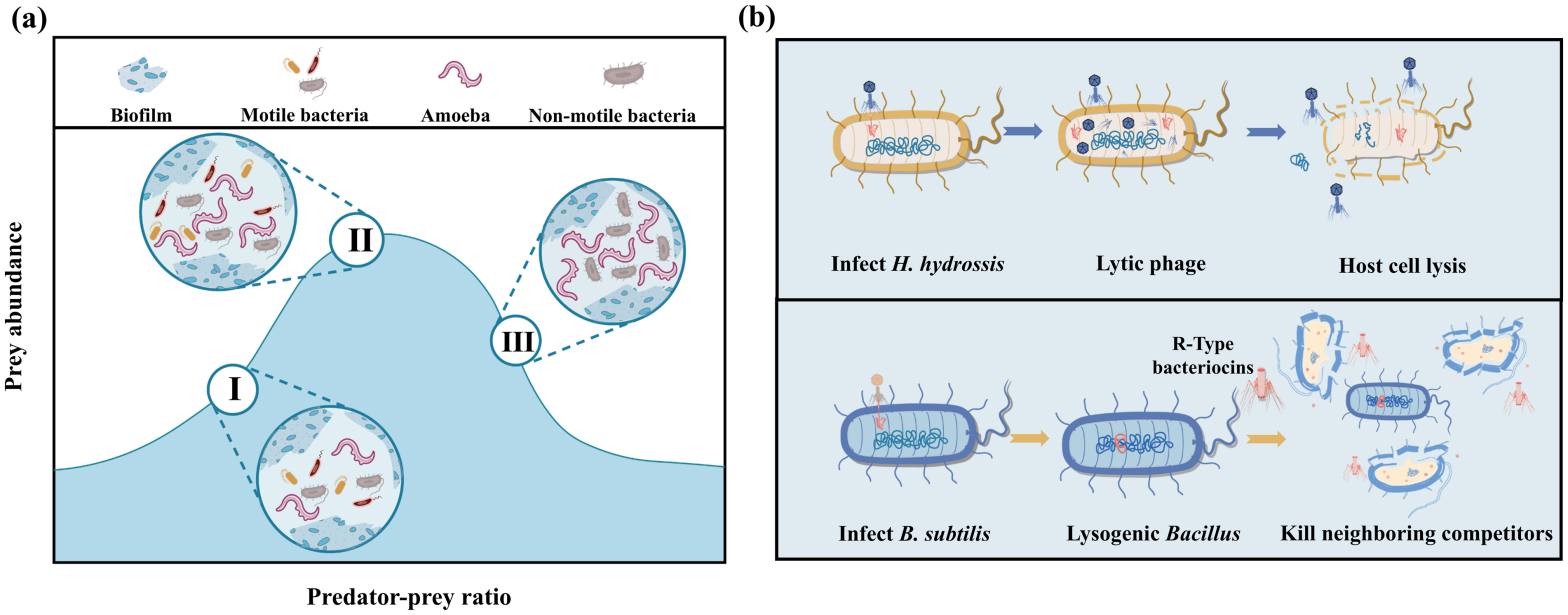
ISRB mechanisms of protozoan/metazoan **(a)** and bacteriophage predation **(b)**.

Protozoa and metazoa exert a sophisticated and effective influence on the sludge reduction via biodegradation and predation processes. These organisms are pivotal in regulating the microbial structure and the associated metabolic functions (Huang et al., 2023). The following are the key biotechnological mechanisms at play:

- Microbial predation: Protozoa, including ciliates, flagellates, and amoebae, along with certain small metazoa like rotifers, contribute to the regulation of microbial communities by preying on excessive bacteria (Hailei et al., 2017). A study reported that nematodes could have a significant impact on the microbial community in biological reactors, where grazing by nematodes was observed to reduce biomass growth by 45% in a biotrickling filter (Klein et al., 2016).
- Correlation between predation and aerobic granule formation: Previous studies, utilizing non-metric multidimensional scaling analysis, have revealed a positive correlation between the presence of protozoa and the size of sludge particles during aerobic granulation (Chan et al., 2021). In the absence of protozoan predators, flocculent sludge could demonstrate diminished compactness and suboptimal settling characteristics, which can impede the formation of granular sludge (Wilén et al., 2018).
- Maximization of bacterial diversity by moderate predation pressure: Optimizing the predation intensity could increase prey diversity with enhanced ecosystem productivity. In high-yielding activated sludge communities, the biomass of protozoa can account for up to 20% of the total biomass (Burian et al., 2022). However, as a medium of top-down control, reducing predation pressure leads to a significant decrease in the richness, evenness, and phylogenetic distinctiveness of the bacterial community. The relationship between predation pressure and prey diversity is not linear but rather resembles a hump-shaped pattern, as shown in Figure 6a.

### 3.4 Bacteriophage lysis

Bacteriophages, as viruses capable of infecting and eradicating specific bacteria, exert a substantial influence on the dynamics of microbial communities and the composition of sludge through intricate infection processes (Carroll-Portillo et al., 2023). Phages can be primarily classified into two distinct types, as shown in Figure 6b. Lytic phages, the first type, are responsible for the lysis of bacteria. They hold significant potential in regulating bacteria with high-growth rates. If competing bacterial cells are lysed by these phages, the intracellular nutrients will be released. This nutrient release serves as a supplementary resource for the growth of neighboring WAS-degrading microbes (Chan et al., 2021). In a particular study, a lytic phage targeting *H. hydrossis* was isolated from the mixed liquor at a wastewater treatment facility. After the addition of the phage at an optimal virus-to-host ratio of 1:1,000, the SVI remarkably decreased from 155 to 105. Interestingly, *H. hydrossis-infecting* phages showed no cross infectivity with other bacteria in activated sludge systems (Kotay et al., 2011).

The second type is the lysogenic phage, which has a symbiotic relationship with slow-growing bacteria and proliferate with the host genome replication accordingly. Under certain conditions, slow-growing bacteria can selectively retain prophage-derived genomic elements, especially under the circumstance that these genes could confer some survival advantages (Gebhart et al., 2012). For example, R-type bacteriocins derived from phage tail-like particles, could eliminate neighboring competitor bacteria to provide slow-growing *Bacillus subtilis* with a competitive edge in the microbial community (Howard-Varona et al., 2017).

In summary, bacteriophages, through their diverse interaction mechanisms with bacteria, play a crucial and multi-faceted role in shaping the ecological balance and functional characteristics of microbial communities in sludge systems, offering new perspectives for the optimization of sludge reduction processes.

### 3.5 Biofilm-based manipulation

**Figure 7:**
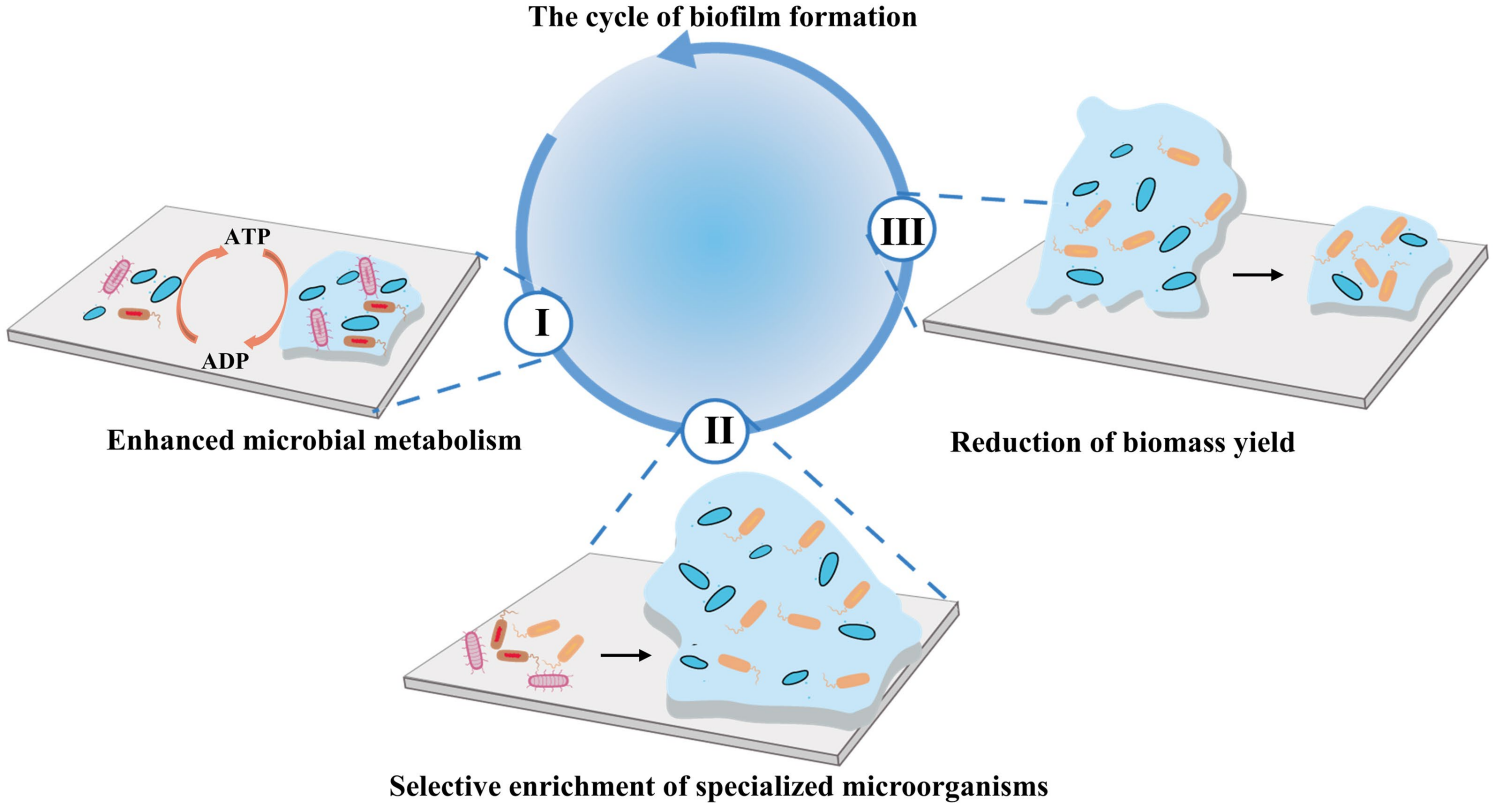
ISRB mechanisms of biofilm-based manipulation.

Biofilms represent highly organized microbial communities where microorganisms adhere to one another and to surfaces, ensconced within a self-synthesized EPS matrix (Wang X.B. et al., 2021). These biofilms exert a multifaceted influence on the sludge reduction process in several ways, where Figure 7 illustrated the cycling process during biofilm formation.

- Enhanced microbial metabolism: In the biofilm environment, microbial cells are in close proximity, which greatly promotes the exchange of genetic materials and metabolic by-products (Xia et al., 2025). Wei et al. (2023) reported that biofilm formation could lead to a remarkable increase in microbial richness. Specifically, the Chao index reached 1,700 on the second day of cultivation. This heightened microbial activity results in more comprehensive degradation of organic matter, which in turn significantly contributes to sludge reduction (Li Y.L. et al., 2022; Li C. et al., 2022; Li W. et al., 2022).
- Selective enrichment of specializers: Biofilms possess the ability to selectively enrich specific microbial species, and the stable microenvironment they establish favors the proliferation of slow-growing microbes and endogenous decay organisms. This promotes microbial self-digestion and renewal, contributing to highly efficient pollutant degradation (Jin et al., 2024). In a sequencing batch biofilm reactor, by adjusting the aeration mode, the removal efficiency of total nitrogen was observed to increase from 58.92 to 94.88%. After biofilm formation, genes related to nitrogen removal, such as amoABC, hao, and napAB, were upregulated. This genetic upregulation redirected the energy flow from WAS accumulation toward nitrogen gas production (Lu et al., 2023).
- Reduction of biomass yield: In a FCS-SBBR bioreactor, *Bacteroidetes* and *Mizugakiibacter*, associated with sludge reduction, were identified as the dominant phylum and genus, respectively, leading to a 49.65% reduction in sludge production (Wang et al., 2018). Compared to suspended growth systems, biofilm-based systems enhance key metabolic processes in the lower sludge layer, such as denitrification, phosphorus release, and anaerobic digestion, thereby accelerating organic matter breakdown and cell lysis while yielding lower biomass production (Freyschmidt and Beier, 2023). This reduced biomass yield is attributed to the slower growth rates and elevated respiratory activity of microorganisms within biofilms, where a greater proportion of metabolic energy is directed toward maintenance and catabolism rather than biomass synthesis (Philipp et al., 2024).

## 4 Application strategies for ISRB

### 4.1 Effects of EPS characteristics

EPS, primarily composed of proteins, carbohydrates, humic substances, nucleic acids, uronic acids, and lipids secreted by microorganisms or released through cell lysis, form a complex polymeric network (Pan M. et al., 2023; To et al., 2020). Approximately 80% of the mass of WAS can be attributed to EPS, which have a profound influence on the viscosity and water-retention capacity of the sludge, thereby exerting a substantial impact on its dewatering performance (Li et al., 2024; Frølund et al., 1996).

Water molecules interact with the EPS structure predominantly via electrostatic forces associated with hydroxyl groups and hydrogen bonds, which could contribute significantly to the overall binding energy of EPS. Consequently, the hydrolysis of EPS can enhance the dewaterability of sludge by reducing the polymer concentration (Neyens et al., 2004). A high content of EPS has been shown to undermine the flocculation and sedimentation performance of sludge, posing challenges for conventional mechanical dewatering processes to attain optimal efficiency (Li Y.L. et al., 2022; Li C. et al., 2022; Li W. et al., 2022). To et al. (2020) discovered that the WAS dewatering performance in the majority of WWTPs is suboptimal (averaging 20 wt%), with TB-EPS playing a decisive role in dewatering efficiency (Li Y.L. et al., 2022; Li C. et al., 2022; Li W. et al., 2022).

Additionally, microbial EPS are well-known for providing a multitude of protective functions, such as cryoprotection, resistance to desiccation, buffering of salinity and pH, which could facilitate the formation of aggregates and biofilms (Nagar et al., 2021). The protective layer formed by EPS can hinder the degradation of organic matter in sludge by microorganisms, thus affecting sludge reduction. Wang S. et al. (2023a) summarized that three major families of biofilm dispersal enzymes, namely glycosidases, proteases, and deoxyribonucleases, which target exopolysaccharides, extracellular proteins, and extracellular DNA respectively, can significantly reduce sludge viscosity, enhance dewatering efficiency, and disrupt the protective functions of EPS by regulating the synthesis and degradation processes. It has also been reported that EPS influence the degradation pathways of pollutants in WWTPs. Shapley additive explanations indicated that soluble EPS, S-EPS_PS_ and S-EPS_PN_, were the most critical factors affecting methane productivity and membrane contamination, respectively. Consequently, these factors impact the “gasification” reaction process of organic carbon (Niu et al., 2024).

### 4.2 Effects of operational parameters

#### 4.2.1 pH

pH stands as a crucial determinant dictating the efficiency of the ISRB process. It exerts its influence by fine-tuning the biochemical and physiological pathways of enzymes, microbial agents, protozoa, metazoa, and bacteriophages (Yan et al., 2023; Liu et al., 2012; Kotay et al., 2011).

Alkaline proteases, secreted by *Bacillus licheniformis*, show a marked increase in production as the pH of the culture medium ascends from 6.0 to 8.0. At a pH of 9.5, these enzymes attain an optimal conformational state, which maximizes their activity. This optimal conformation facilitates enhanced substrate binding and catalytic efficiency, thereby expediting the relevant biochemical reactions (Zhang J.H. et al., 2023). Conversely, cellulases sourced from *Trichoderma reesei* display peak activity at pH 6.0, where the enzyme’s tertiary structure is stabilized, conducive to the efficient hydrolysis of cellulose (Juhász et al., 2004).

Microbial agents such as *Bacillus amyloliquefacien* thrive in a neutral pH environment. In such conditions, their metabolic activities, including the breakdown of complex organic compounds, are optimized. This is primarily due to the pH-sensitive regulatory networks that govern their metabolic functions (Zhang X. et al., 2023).

Amoebas and rotifers exhibit vigorous proliferation within distinct pH ranges. Amoebas proliferate optimally at pH values between 5.9 and 7.1, while rotifers at pH values from 8.2 to 9.4. These pH ranges are essential for maintaining membrane integrity and osmoregulation, which are critical for their effective predation of harmful bacteria (Ramirez et al., 2014; Ergönül et al., 2016).

Bacteriophages, known for their ability to infect sludge foaming and bulking filamentous bacteria, demonstrate remarkable stability and infectivity within a pH range of 5–8. In this range, the adsorption of the phage onto the host bacteria and the subsequent injection of its DNA are highly efficient (Kotay et al., 2011).

However, the application of pH control in sludge reduction processes faces significant challenges. The biological entities involved in the ISRB process have widely varying optimal pH values. This disparity makes it extremely difficult to maintain a single, uniform pH that can optimize all relevant biological and biochemical processes. Additionally, the inherent heterogeneity of sludge can lead to local pH variations. These variations result in sub-optimal conditions for different microbial and enzymatic activities, thereby impeding the overall efficiency of the sludge reduction process (Guimet et al., 2007; van den Berg et al., 2020).

To address these challenges, the development of a prioritization model could be a viable strategy. Such a model would enable a comprehensive evaluation of the impact of these factors on the overall performance of the system (Zhai et al., 2024). Consequently, it would assist in formulating optimized pH control strategies for more effective sludge reduction and biodegradation.

#### 4.2.2 Microbial interactions

In the activated sludge (AS) system, the microbial community exhibits remarkable dynamism and complexity, encompassing a rich array of microorganisms, including bacteria, protozoa, fungi, and other taxa. This intricate microbial community plays an indispensable role in modulating metabolic pathways and interactions during the degradation of complex organic matter, where functional diversity and stability are pivotal determinants of the efficiency of these degradation processes.

Notably, the microbial communities belonging to the phyla Bacteroidetes and Proteobacteria collectively constitute over 50% of the AS system, with genera such as *Fluviicola*, *Thiobacillus*, *Sphingopyxis*, and *Thermomonas* being predominant (Tian and Wang, 2020). Bacteroidota are regarded as prime candidates for degrading high-molecular-weight organic matter, such as proteins and carbohydrates. Proteobacteria, on the other hand, are crucial for anaerobic hydrolysis and acidification, responsible for the degradation of organic matter and nitrogen/phosphorus removal. The rapid metabolism and reproduction of both phyla make substantial contributions to sludge reduction. A microbial community with such a structure can enhance sludge reduction efficiency by up to 50%, in stark contrast to monoculture approaches, which achieve significantly lower reduction rates due to the absence of complementary metabolic activities (Bian et al., 2020).

Furthermore, AS systems harbor a substantial number of prokaryotic viruses, with concentrations ranging from 10 to 1,000 times higher than those in natural environments (Chen et al., 2021). These phages are closely associated with functional microorganisms involved in processes like nitrogen and phosphorus removal. Phages are typically characterized by their broad host ranges and can be applied through a phage cocktail strategy. This approach involves the targeted combination of multiple phages to enhance bacterial suppression and meet the specific requirements of different bacterial strains present in wastewater treatment plants.

Introducing exogenous microorganisms is a promising approach for improving the performance of AS systems. However, the most significant limitation lies in the difficulty of maintaining an optimal microbial community that can function consistently under diverse environmental conditions. Additionally, predicting and managing the interactions between introduced microbial populations and the indigenous sludge microbial communities is a complex task. Competition or antagonistic effects between these populations may impede the overall efficacy of the microbial agents, thereby posing challenges to the efficient operation of the AS system.

#### 4.2.3 Aeration mode

Oxygen conditions exert a direct and profound influence on the overall efficiency of hydrolytic enzymes, such as cellulases and proteases (Kumari et al., 2024). Aerobic microorganisms, including *Pseudomonas*, *Bacillus*, and Actinobacteria, display distinct growth and metabolic patterns under different dissolved oxygen (DO) levels. In high-oxygen environments, these microbes are highly active, effectively facilitating the decomposition of organic matter in sludge. On the other hand, anaerobic conditions favor hydrolysis and fermentation, leading to the establishment of a lower redox potential (approximately −350 mV), which in turn enriches anaerobic hydrolytic bacteria and enhances downstream sludge reduction (Zhou et al., 2023). Although aerobic conditions accelerate the breakdown of organic matter and stimulate microbial activity, they are energy-intensive and may not ensure long-term sludge reduction. In contrast, anaerobic environments may result in incomplete degradation of organic matter, producing hard-to-degrade intermediate products such as sulfides (Jørgensen et al., 2019).

In the context of ISRB, the aeration mode and its duration, which directly impact DO concentrations, are of paramount importance. Direct aeration is more efficient in introducing oxygen, supporting the growth of aerobic microbial populations. This, in turn, enhances enzymatic activity and leads to a higher degradation rate of organic matter in the sludge. For instance, Guo et al. reported that when DO levels were increased from 2 to 6 mg/L, sludge reduction reached 25% (Guo et al., 2020). Intermittent aeration, on the other hand, often creates localized oxygen-deficient zones. These zones promote anaerobic microbial processes, which, although slower, contribute to sludge reduction through pathways such as fermentation and methanogenesis. However, prolonged aeration can cause overoxidation and reduce sludge reduction efficiency by decreased microbial diversity. For example, the proportion of Proteobacteria increased significantly from 0.08 to 19.9 and 22.6% when the DO was decreased from 4 mg/L to 2 mg/L and 0.8 mg/L, respectively. This clearly demonstrates that precise optimization of aeration parameters is crucial for maximizing sludge reduction (Izadi et al., 2021).

### 4.3 Effects of nutrient supply

#### 4.3.1 Key nutrients

In the ISRB process, key nutrients including carbon, nitrogen, and phosphorus play a pivotal role. They serve as the foundation for microbial growth and metabolism, exerting a direct influence on the overall stability of the system (Guo et al., 2020).

Denitrification systems employing diverse carbon sources exhibit significant disparities in macroscopic carbon utilization efficiency and microscopic microbial metabolism. Long term bioreactor operation and kinetic studies indicated that the reactor utilizing acetate achieved the highest denitrification rates, while the one utilizing methanol produced the least sludge after the acclimation period (Pan Y. et al., 2023). In addition, glyoxylate acts as a crucial intermediate in the glyoxylate cycle. This cycle enables bacteria, fungi, and plants to harness two-carbon compounds such as acetate for energy production and biosynthesis under carbon-limited conditions. Disrupting this cycle in bacteria could lead to the loss of respiratory activity, biofilm formation capacity, and essential energy supply. This is primarily attributed to imbalanced material and energy metabolism, the collapse of the antioxidant system and reduced extracellular polymer synthesis. Nevertheless, the excessive addition of carbon sources can trigger over-active microbial activity, thereby increasing the total sludge volume (Qi et al., 2023).

Nitrogen sources, such as ammonia and nitrate, are indispensable for the synthesis of amino acids and proteins, which have a profound impact on the community structure, and metabolic pathways of AS microorganisms. With a high C/N ratio, an excess of carbon but a deficiency of nitrogen forces microorganisms to rely on heterotrophic metabolism for energy. However, the lack of nitrogen constrains cell growth and division. Conversely, at a low C/N ratio, although nitrogen is abundant, carbon deficiency restricts microbial growth, resulting in reduced metabolic rates and inferior sludge reduction efficiency (Wang S. et al., 2023b).

Phosphorus, a vital component of adenosine triphosphate (ATP) and cell membranes, is crucial for regulating cellular activities and metabolic processes. Different forms of phosphorus, such as phosphate, superphosphate, and phytate, could modify metabolic pathways, including glutathione metabolism, cysteine metabolism, and galactose metabolism. Nevertheless, an increase in phosphorus levels can stimulate algae growth, especially when phosphorus concentrations in aquatic environments rise, leading to eutrophication and a deterioration of water quality. Additionally, excessive phosphorus can disrupt the decoupling process, thereby affecting system stability and efficiency (Kang et al., 2023).

#### 4.3.2 Trace nutrients

Trace nutrients, including iron, zinc, nickel, and cobalt, primarily influence sludge reduction by modulating enzyme activity and microbial metabolic functions. Iron acts as a cofactor for redox enzymes, such as iron–sulfur proteins and ferredoxin (Rai et al., 2021). Its presence is crucial as it participates in electron transfer processes, which are fundamental for many microbial redox reactions within the AS system. Zinc, characterized by its strong proclivity to form tetrahedral complexes, is a key component of proteins associated with DNA and RNA synthesis. Transcription factors, reverse transcriptase, and RNA polymerases rely on zinc for their proper functioning (Rai et al., 2021). These proteins are essential for genetic information transfer and regulation in microorganisms. Cobalt is intricately involved in the synthesis of vitamin B12, an essential coenzyme in numerous anaerobic microbial metabolic pathways (Stabler, 2020).

The application of trace nutrients in sludge reduction demands well-thought-out strategies. In anaerobic digestion systems, maintaining adequate levels of nickel and cobalt is imperative to sustain stable microbial metabolism. However, an increase in metal solubility can potentially intensify microbial toxicity (Zadeh et al., 2022). This is a critical concern as excessive metal concentrations can disrupt microbial cell membranes, inhibit enzyme activities, and ultimately impede the sludge reduction process.

Furthermore, the application of trace nutrients in sludge reduction still faces several challenges. These elements are often costly and susceptible to loss during wastewater treatment, leading to increased operational expenses. Their bioavailability is limited, as they tend to precipitate or exist in forms that are difficult for microorganisms to utilize (Pereira et al., 2015). Interactions between different elements, including antagonistic and synergistic effects, may further influence their effectiveness. To address these challenges, optimizing treatment processes by incorporating chelating agents to enhance bioavailability, along with precise control of micronutrient concentrations, can improve sludge reduction efficiency while minimizing costs (Bardi et al., 2023).

## 5 Discussion and conclusion

This comprehensive review systematically synthesizes the latest advancements in biotechnological strategies for *in situ* sludge minimization within wastewater treatment systems. Particular emphasis is placed on the underlying mechanisms and application tactics of various ISRB methods, including enzymatic hydrolysis, microbial inoculation, protozoan/metazoan predation, bacteriophage lysis, and biofilm-based strategies.

The findings presented herein indicate that ISRBs confer several remarkable advantages over traditional sludge reduction techniques. These include lower energy consumption, reduced chemical usage, and minimized environmental risks. ISRB has demonstrated promising performance and engineering feasibility under both laboratory and pilot-scale conditions, with representative technologies such as anaerobic membrane bioreactors (AnMBRs) and anaerobic ammonium oxidation (Anammox) (Su et al., 2023; Rong et al., 2023). However, its effectiveness is strongly influenced by key operational parameters, including pH, microbial community structure, aeration mode, and nutrient availability. Despite the remarkable progress achieved in ISRB relevant studies, several limitations outlined still pose obstacles to its widespread application in large-scale wastewater treatment systems. Enzymatic treatment has demonstrated substantial potential in decomposing complex macromolecules and enhancing sludge biodegradability. However, challenges related to enzyme stability and high production costs remain unresolved. Similarly, microbial inoculants can effectively optimize the composition of the sludge microbial community and enhance process stability. Yet, their long-term effectiveness is frequently hindered by intricate interspecies interactions. Strategies based on predation by protozoa, metazoa, and bacteriophages represent innovative biocontrol methods for regulating microbial populations. However, further in-depth studies will be required to clarify the predator–prey dynamics and determine the optimal application conditions.

Looking ahead, future research endeavors should focus on integrating multiple ISRB strategies to generate synergistic effects that improve sludge reduction efficiency and stability. The development of customized microbial consortia, genetic engineering of microbial strains to enhance degradation capabilities, and the application of emerging technologies such as bioelectrochemical systems and advanced biofilm reactors offer promising avenues for research and development. Moreover, conducting comprehensive whole-life-cycle assessments to address the environmental and economic impacts of these technologies is crucial for evaluating their contribution to carbon neutrality.

## Glossary

WWTPs: wastewater treatment plants
ISRB: *in situ* sludge reduction biotechnology
WAS: waste activated sludge
TSR: traditional sludge reduction
ISR: *in situ* sludge reduction
EPS: extracellular polymeric substances
CST: capillary suction time
SNADF: simultaneous partial nitrification anammox denitrification and fermentation
MLSS: mixed liquor suspended solids
COD: chemical oxygen demand
CFU: colony-forming unit
SVI: sludge volume index
FCS-SBBR: sequencing batch biofilm reactor with composite floating spherical carries
TB-EPS: tightly bound extracellular polymeric substances
S-EPS_PS_: soluble extracellular polymeric substances polysaccharide
S-EPS_PN_: soluble extracellular polymeric substances protein
pH: potential of hydrogen
DNA: deoxyribonucleic acid
AS: activated sludge
DO: dissolved oxygen
ATP: adenosine triphosphate
RNA: ribonucleic acid
AnMBRs: anaerobic membrane bioreactors
Anammox: anaerobic ammonium oxidation
